# 1-[Bis(4-fluoro­phen­yl)meth­yl]-4-[2-(2-methyl­phen­oxy)eth­yl]piperazine

**DOI:** 10.1107/S1600536812010744

**Published:** 2012-03-17

**Authors:** Zhao-Hui Dai, Yan Zhong, Bin Wu

**Affiliations:** a School of Pharmacy, Nanjing Medical University, Hanzhong Road No. 140 Nanjing, Nanjing 210029, People’s Republic of China; b School of Chemistry and Chemical Engineering, Southeast University, Sipailou No. 2 Nanjing, Nanjing 210096, People’s Republic of China

## Abstract

In the title mol­ecule, C_26_H_28_F_2_N_2_O, the piperazine ring adopts a chair conformation, with the N-bonded substituents in equatorial orientations. The dihedral angle between the fluoro­benzene rings is 69.10 (15).

## Related literature

For related structures and background to 1-(bis­(4-fluoro­phen­yl)meth­yl)piperazine derivatives, see: Wu *et al.* (2008 ▶); Dayananda *et al.* (2012 ▶); Zhong *et al.* (2011 ▶).
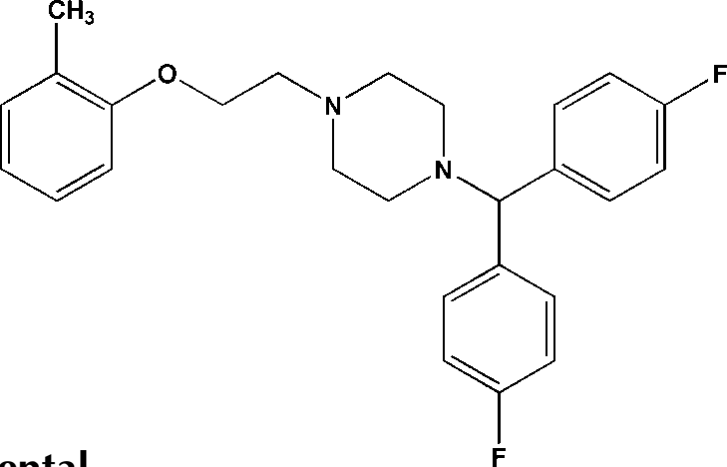


## Experimental

### 

#### Crystal data

C_26_H_28_F_2_N_2_O
*M* *_r_* = 422.50
Monoclinic, 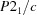

*a* = 10.021 (2) Å
*b* = 15.203 (3) Å
*c* = 15.868 (3) Å
β = 100.54 (3)°
*V* = 2376.7 (8) Å^3^
*Z* = 4
Mo *K*α radiation
μ = 0.08 mm^−1^
*T* = 293 K
0.30 × 0.20 × 0.10 mm

#### Data collection

Enraf–Nonious CAD-4 diffractometer
Absorption correction: ψ scan (North *et al.*, 1968) ▶ *T*_min_ = 0.976, *T*_max_ = 0.992
4618 measured reflections
4355 independent reflections
2404 reflections with *I* > 2σ(*I*)
*R*_int_ = 0.040
3 standard reflections every 200 reflections intensity decay: 1%

#### Refinement

*R*[*F* ^2^ > 2σ(*F* ^2^)] = 0.063
*wR*(*F* ^2^) = 0.191
*S* = 1.03
4355 reflections
281 parameters
H-atom parameters constrained
Δρ_max_ = 0.16 e Å^−3^
Δρ_min_ = −0.19 e Å^−3^

### 

Data collection: *CAD-4 EXPRESS* (Enraf–Nonius, 1994 ▶); cell refinement: *CAD-4 EXPRESS*; data reduction: *XCAD4* (Harms & Wocadlo, 1995 ▶); program(s) used to solve structure: *SHELXS97* (Sheldrick, 2008 ▶); program(s) used to refine structure: *SHELXL97* (Sheldrick, 2008 ▶); molecular graphics: *SHELXTL* (Sheldrick, 2008 ▶); software used to prepare material for publication: *SHELXL97*.

## Supplementary Material

Crystal structure: contains datablock(s) I, global. DOI: 10.1107/S1600536812010744/hb6677sup1.cif

Structure factors: contains datablock(s) I. DOI: 10.1107/S1600536812010744/hb6677Isup2.hkl

Supplementary material file. DOI: 10.1107/S1600536812010744/hb6677Isup3.cml

Additional supplementary materials:  crystallographic information; 3D view; checkCIF report

## Figures and Tables

**Fig. 1.**
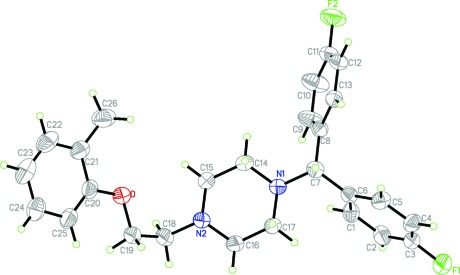
The molecular structure of (I) with displacement ellipsoids for non-H drawn at 70% probability level.

**Fig. 2.**
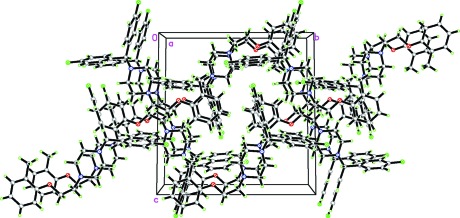
Packing diagram of the title compound.

## supplementary crystallographic information

### Comment

As a continuation of our study of 1-(bis(4-fluorophenyl)methyl)piperazine derivatives (Wu *et al.*, 2008; Zhong *et al.*, 2011), we present here the title compound (I). In (I) (Fig. 1), all bond lengths and angles are normal and correspond to those observed in related compounds (Zhong *et al.*, 2011). The piperazine ring adopts a chair conformation with puchering parameters Q = 0.591 (3), Theta = 1.7 (3), Phi = 333 (6). The dihedral angle between the fluorobenzene rings is 69.10 (15).

### Experimental

A mixture of 1-(2-bromoethoxy)-2-methylbenzene (10 mmol), 1-(bis(4-fluorophenyl)methyl)piperazine (15 mmol) and triethylamine (5 ml) were mixed along with 40 ml acetonitrile and then refluxed for about 24 h. The progress of the reaction was monitored by TLC. After confirming that the reaction was completed, the solvent was removed under reduced pressure. The resultant mixture was cooled. The solid, 1-(bis(4-fluorophenyl)methyl)-4-(2-(2-methylphenoxy)ethyl)piperazine obtained was filtered and was recrystallized from ethanol. The colorless blocks of the title compound were grown in ethanol by a slow evaporation at room temperature.

### Refinement

All hydrogen atoms were positioned geometrically with C—H distances ranging from 0.93 Å to 0.98 Å and refined as riding on their parent atoms with *U*ĩso~(H) = 1.2 or 1.5*U*~eq~ of the carrier atom.

### Crystal data

**Table:**

C_26_H_28_F_2_N_2_O	*F*(000) = 896
*M**_r_* = 422.50	*D*_x_ = 1.181 Mg m^−^^3^
Monoclinic, *P*2_1_/*c*	Mo *K*α radiation, λ = 0.71073 Å
*a* = 10.021 (2) Å	Cell parameters from 25 reflections
*b* = 15.203 (3) Å	θ = 10–13°
*c* = 15.868 (3) Å	µ = 0.08 mm^−^^1^
β = 100.54 (3)°	*T* = 293 K
*V* = 2376.7 (8) Å^3^	Block, colorless
*Z* = 4	0.30 × 0.20 × 0.10 mm

### Data collection

**Table:**

Enraf–Nonious CAD-4 diffractometer	2404 reflections with *I* > 2σ(*I*)
Radiation source: fine-focus sealed tube	*R*_int_ = 0.040
Graphite monochromator	θ_max_ = 25.4°, θ_min_ = 1.9°
ω/2θ scans	*h* = 0→12
Absorption correction: ψ scan (North *et al.*, 1968)	*k* = 0→18
*T*_min_ = 0.976, *T*_max_ = 0.992	*l* = −19→18
4618 measured reflections	3 standard reflections every 200 reflections
4355 independent reflections	intensity decay: 1%

### Refinement

**Table:**

Refinement on *F*^2^	Secondary atom site location: difference Fourier map
Least-squares matrix: full	Hydrogen site location: inferred from neighbouring sites
*R*[*F*^2^ > 2σ(*F*^2^)] = 0.063	H-atom parameters constrained
*wR*(*F*^2^) = 0.191	*w* = 1/[σ^2^(*F*_o_^2^) + (0.1*P*)^2^] where *P* = (*F*_o_^2^ + 2*F*_c_^2^)/3
*S* = 1.03	(Δ/σ)_max_ < 0.001
4355 reflections	Δρ_max_ = 0.16 e Å^−^^3^
281 parameters	Δρ_min_ = −0.19 e Å^−^^3^
0 restraints	Extinction correction: *SHELXL97* (Sheldrick, 2008), Fc^*^=kFc[1+0.001xFc^2^λ^3^/sin(2θ)]^-1/4^
Primary atom site location: structure-invariant direct methods	Extinction coefficient: 0.015 (2)

### Special details

**Table:**

Geometry. All e.s.d.'s (except the e.s.d. in the dihedral angle between two l.s. planes) are estimated using the full covariance matrix. The cell e.s.d.'s are taken into account individually in the estimation of e.s.d.'s in distances, angles and torsion angles; correlations between e.s.d.'s in cell parameters are only used when they are defined by crystal symmetry. An approximate (isotropic) treatment of cell e.s.d.'s is used for estimating e.s.d.'s involving l.s. planes.
Refinement. Refinement of *F*^2^ against ALL reflections. The weighted *R*-factor *wR* and goodness of fit *S* are based on *F*^2^, conventional *R*-factors *R* are based on *F*, with *F* set to zero for negative *F*^2^. The threshold expression of *F*^2^ > σ(*F*^2^) is used only for calculating *R*-factors(gt) *etc*. and is not relevant to the choice of reflections for refinement. *R*-factors based on *F*^2^ are statistically about twice as large as those based on *F*, and *R*- factors based on ALL data will be even larger.

### Fractional atomic coordinates and isotropic or equivalent isotropic displacement parameters (Å^2^)

**Table:**

	*x*	*y*	*z*	*U*_iso_*/*U*_eq_	
O	0.7306 (2)	1.14171 (14)	0.42351 (12)	0.0810 (7)	
N1	0.7468 (2)	0.84241 (14)	0.22937 (13)	0.0581 (6)	
F1	0.8567 (2)	0.43605 (12)	0.19160 (11)	0.1000 (7)	
C1	0.7161 (3)	0.65379 (19)	0.18894 (18)	0.0643 (8)	
H1A	0.6365	0.6807	0.1978	0.077*	
N2	0.7479 (2)	0.95279 (15)	0.37811 (14)	0.0686 (7)	
F2	0.5198 (2)	0.89761 (15)	−0.16486 (11)	0.1113 (7)	
C2	0.7277 (3)	0.5639 (2)	0.19660 (18)	0.0721 (9)	
H2A	0.6576	0.5304	0.2111	0.087*	
C3	0.8442 (4)	0.5253 (2)	0.18250 (17)	0.0708 (8)	
C4	0.9473 (3)	0.5721 (2)	0.1593 (2)	0.0756 (9)	
H4A	1.0247	0.5441	0.1482	0.091*	
C5	0.9339 (3)	0.6618 (2)	0.15294 (18)	0.0681 (8)	
H5A	1.0042	0.6945	0.1376	0.082*	
C6	0.8195 (3)	0.70541 (18)	0.16848 (15)	0.0552 (7)	
C7	0.8082 (3)	0.80441 (18)	0.16018 (16)	0.0561 (7)	
H7A	0.9001	0.8284	0.1652	0.067*	
C8	0.7296 (3)	0.82920 (17)	0.07345 (16)	0.0546 (7)	
C9	0.5904 (3)	0.8252 (3)	0.05368 (19)	0.0904 (11)	
H9A	0.5427	0.8064	0.0954	0.108*	
C10	0.5197 (3)	0.8480 (3)	−0.0254 (2)	0.1014 (13)	
H10A	0.4253	0.8455	−0.0369	0.122*	
C11	0.5892 (3)	0.8743 (2)	−0.08663 (18)	0.0724 (9)	
C12	0.7263 (3)	0.87824 (19)	−0.07114 (18)	0.0685 (8)	
H12A	0.7727	0.8961	−0.1138	0.082*	
C13	0.7958 (3)	0.85550 (17)	0.00850 (17)	0.0597 (7)	
H13A	0.8901	0.8578	0.0191	0.072*	
C14	0.7401 (3)	0.93889 (19)	0.22319 (18)	0.0713 (8)	
H14A	0.6883	0.9558	0.1678	0.086*	
H14B	0.8311	0.9626	0.2278	0.086*	
C15	0.6743 (3)	0.9766 (2)	0.29339 (17)	0.0745 (9)	
H15A	0.6709	1.0402	0.2882	0.089*	
H15B	0.5818	0.9552	0.2867	0.089*	
C16	0.7577 (4)	0.85698 (18)	0.38358 (18)	0.0739 (9)	
H16A	0.6674	0.8320	0.3782	0.089*	
H16B	0.8091	0.8403	0.4391	0.089*	
C17	0.8258 (3)	0.82076 (18)	0.31398 (16)	0.0643 (8)	
H17A	0.9163	0.8454	0.3195	0.077*	
H17B	0.8342	0.7574	0.3199	0.077*	
C18	0.6835 (4)	0.9899 (2)	0.44565 (19)	0.0815 (9)	
H18A	0.6856	0.9462	0.4904	0.098*	
H18B	0.5891	1.0019	0.4219	0.098*	
C19	0.7477 (4)	1.07238 (19)	0.48507 (19)	0.0823 (10)	
H19A	0.7063	1.0889	0.5335	0.099*	
H19B	0.8436	1.0624	0.5059	0.099*	
C20	0.7721 (3)	1.2243 (2)	0.4525 (2)	0.0683 (8)	
C21	0.7494 (3)	1.2900 (2)	0.3902 (2)	0.0737 (9)	
C22	0.7859 (4)	1.3752 (2)	0.4162 (3)	0.0940 (11)	
H22A	0.7715	1.4205	0.3762	0.113*	
C23	0.8430 (4)	1.3944 (2)	0.5001 (3)	0.0995 (12)	
H23A	0.8660	1.4521	0.5160	0.119*	
C24	0.8657 (3)	1.3293 (2)	0.5596 (2)	0.0859 (10)	
H24A	0.9042	1.3423	0.6161	0.103*	
C25	0.8314 (3)	1.2432 (2)	0.5358 (2)	0.0778 (9)	
H25A	0.8484	1.1982	0.5761	0.093*	
C26	0.6884 (4)	1.2689 (3)	0.2986 (2)	0.1020 (12)	
H26A	0.6800	1.3219	0.2651	0.153*	
H26B	0.6003	1.2432	0.2962	0.153*	
H26C	0.7459	1.2281	0.2759	0.153*	

### Atomic displacement parameters (Å^2^)

**Table:**

	*U*^11^	*U*^22^	*U*^33^	*U*^12^	*U*^13^	*U*^23^
O	0.1193 (18)	0.0623 (13)	0.0637 (13)	0.0113 (12)	0.0232 (12)	0.0065 (10)
N1	0.0634 (14)	0.0592 (15)	0.0511 (13)	0.0048 (11)	0.0091 (11)	0.0049 (10)
F1	0.1421 (18)	0.0689 (13)	0.0948 (14)	0.0127 (11)	0.0372 (13)	0.0029 (10)
C1	0.0565 (17)	0.066 (2)	0.0750 (19)	−0.0016 (15)	0.0229 (15)	−0.0084 (15)
N2	0.0968 (18)	0.0572 (15)	0.0523 (13)	0.0053 (13)	0.0147 (13)	0.0038 (11)
F2	0.1128 (16)	0.1552 (19)	0.0604 (11)	0.0259 (14)	0.0010 (11)	0.0235 (12)
C2	0.083 (2)	0.068 (2)	0.070 (2)	−0.0132 (17)	0.0276 (17)	−0.0105 (15)
C3	0.100 (2)	0.0559 (19)	0.0590 (18)	0.0070 (18)	0.0206 (17)	0.0017 (14)
C4	0.076 (2)	0.075 (2)	0.079 (2)	0.0236 (17)	0.0225 (17)	0.0128 (17)
C5	0.0527 (17)	0.077 (2)	0.077 (2)	0.0093 (15)	0.0180 (15)	0.0144 (16)
C6	0.0532 (16)	0.0625 (18)	0.0507 (15)	0.0007 (14)	0.0118 (13)	−0.0001 (13)
C7	0.0465 (15)	0.0654 (18)	0.0574 (16)	−0.0024 (13)	0.0121 (13)	0.0024 (13)
C8	0.0532 (16)	0.0569 (17)	0.0560 (16)	0.0008 (13)	0.0158 (13)	0.0014 (13)
C9	0.0542 (19)	0.158 (3)	0.0602 (19)	−0.005 (2)	0.0144 (15)	0.022 (2)
C10	0.0563 (19)	0.179 (4)	0.067 (2)	0.005 (2)	0.0075 (17)	0.024 (2)
C11	0.081 (2)	0.085 (2)	0.0493 (17)	0.0114 (18)	0.0069 (16)	0.0079 (15)
C12	0.085 (2)	0.069 (2)	0.0567 (18)	−0.0005 (16)	0.0274 (16)	0.0079 (14)
C13	0.0613 (17)	0.0570 (17)	0.0652 (18)	−0.0030 (13)	0.0235 (15)	0.0035 (14)
C14	0.091 (2)	0.063 (2)	0.0593 (17)	0.0127 (16)	0.0123 (16)	0.0119 (14)
C15	0.099 (2)	0.0620 (18)	0.0628 (19)	0.0173 (17)	0.0166 (17)	0.0043 (15)
C16	0.107 (3)	0.0568 (18)	0.0571 (17)	0.0023 (16)	0.0118 (17)	0.0059 (14)
C17	0.082 (2)	0.0520 (16)	0.0561 (17)	0.0041 (14)	0.0047 (15)	0.0035 (13)
C18	0.119 (3)	0.069 (2)	0.0622 (18)	0.0002 (19)	0.0302 (18)	0.0009 (15)
C19	0.128 (3)	0.062 (2)	0.0609 (18)	0.0072 (18)	0.0258 (19)	0.0022 (15)
C20	0.071 (2)	0.0590 (19)	0.081 (2)	0.0123 (15)	0.0305 (17)	0.0012 (17)
C21	0.0648 (19)	0.073 (2)	0.089 (2)	0.0119 (16)	0.0293 (17)	0.0168 (18)
C22	0.083 (2)	0.072 (3)	0.134 (3)	0.0067 (19)	0.038 (2)	0.030 (2)
C23	0.078 (2)	0.066 (2)	0.157 (4)	0.0027 (19)	0.030 (3)	−0.001 (3)
C24	0.067 (2)	0.077 (3)	0.114 (3)	0.0067 (17)	0.0155 (19)	−0.011 (2)
C25	0.082 (2)	0.061 (2)	0.092 (2)	0.0117 (16)	0.0218 (19)	0.0016 (17)
C26	0.107 (3)	0.109 (3)	0.096 (3)	0.018 (2)	0.034 (2)	0.037 (2)

### Geometric parameters (Å, º)

**Table:**

O—C20	1.375 (3)	C12—H12A	0.9300
O—C19	1.426 (3)	C13—H13A	0.9300
N1—C17	1.466 (3)	C14—C15	1.508 (4)
N1—C14	1.471 (3)	C14—H14A	0.9700
N1—C7	1.472 (3)	C14—H14B	0.9700
F1—C3	1.367 (3)	C15—H15A	0.9700
C1—C2	1.375 (4)	C15—H15B	0.9700
C1—C6	1.385 (4)	C16—C17	1.505 (4)
C1—H1A	0.9300	C16—H16A	0.9700
N2—C15	1.456 (3)	C16—H16B	0.9700
N2—C18	1.462 (4)	C17—H17A	0.9700
N2—C16	1.461 (3)	C17—H17B	0.9700
F2—C11	1.355 (3)	C18—C19	1.493 (4)
C2—C3	1.362 (4)	C18—H18A	0.9700
C2—H2A	0.9300	C18—H18B	0.9700
C3—C4	1.360 (4)	C19—H19A	0.9700
C4—C5	1.373 (4)	C19—H19B	0.9700
C4—H4A	0.9300	C20—C25	1.376 (4)
C5—C6	1.385 (4)	C20—C21	1.394 (4)
C5—H5A	0.9300	C21—C22	1.389 (5)
C6—C7	1.513 (4)	C21—C26	1.505 (5)
C7—C8	1.503 (3)	C22—C23	1.380 (5)
C7—H7A	0.9800	C22—H22A	0.9300
C8—C9	1.374 (4)	C23—C24	1.357 (5)
C8—C13	1.383 (3)	C23—H23A	0.9300
C9—C10	1.368 (4)	C24—C25	1.388 (4)
C9—H9A	0.9300	C24—H24A	0.9300
C10—C11	1.356 (4)	C25—H25A	0.9300
C10—H10A	0.9300	C26—H26A	0.9600
C11—C12	1.352 (4)	C26—H26B	0.9600
C12—C13	1.371 (4)	C26—H26C	0.9600
			
C20—O—C19	117.0 (2)	N2—C15—C14	111.8 (2)
C17—N1—C14	107.2 (2)	N2—C15—H15A	109.3
C17—N1—C7	111.4 (2)	C14—C15—H15A	109.3
C14—N1—C7	111.2 (2)	N2—C15—H15B	109.3
C2—C1—C6	122.0 (3)	C14—C15—H15B	109.3
C2—C1—H1A	119.0	H15A—C15—H15B	107.9
C6—C1—H1A	119.0	N2—C16—C17	110.8 (2)
C15—N2—C18	111.3 (2)	N2—C16—H16A	109.5
C15—N2—C16	108.7 (2)	C17—C16—H16A	109.5
C18—N2—C16	112.0 (2)	N2—C16—H16B	109.5
C3—C2—C1	118.4 (3)	C17—C16—H16B	109.5
C3—C2—H2A	120.8	H16A—C16—H16B	108.1
C1—C2—H2A	120.8	N1—C17—C16	110.4 (2)
C4—C3—C2	122.4 (3)	N1—C17—H17A	109.6
C4—C3—F1	119.2 (3)	C16—C17—H17A	109.6
C2—C3—F1	118.4 (3)	N1—C17—H17B	109.6
C3—C4—C5	118.2 (3)	C16—C17—H17B	109.6
C3—C4—H4A	120.9	H17A—C17—H17B	108.1
C5—C4—H4A	120.9	N2—C18—C19	114.7 (3)
C4—C5—C6	122.3 (3)	N2—C18—H18A	108.6
C4—C5—H5A	118.8	C19—C18—H18A	108.6
C6—C5—H5A	118.8	N2—C18—H18B	108.6
C5—C6—C1	116.7 (3)	C19—C18—H18B	108.6
C5—C6—C7	120.8 (2)	H18A—C18—H18B	107.6
C1—C6—C7	122.5 (2)	O—C19—C18	110.2 (3)
N1—C7—C8	111.3 (2)	O—C19—H19A	109.6
N1—C7—C6	111.1 (2)	C18—C19—H19A	109.6
C8—C7—C6	110.3 (2)	O—C19—H19B	109.6
N1—C7—H7A	108.0	C18—C19—H19B	109.6
C8—C7—H7A	108.0	H19A—C19—H19B	108.1
C6—C7—H7A	108.0	O—C20—C25	124.2 (3)
C9—C8—C13	116.6 (3)	O—C20—C21	114.6 (3)
C9—C8—C7	122.6 (2)	C25—C20—C21	121.2 (3)
C13—C8—C7	120.8 (2)	C22—C21—C20	117.2 (3)
C10—C9—C8	122.2 (3)	C22—C21—C26	121.7 (3)
C10—C9—H9A	118.9	C20—C21—C26	121.1 (3)
C8—C9—H9A	118.9	C23—C22—C21	121.6 (3)
C11—C10—C9	118.9 (3)	C23—C22—H22A	119.2
C11—C10—H10A	120.5	C21—C22—H22A	119.2
C9—C10—H10A	120.5	C24—C23—C22	120.3 (4)
C12—C11—F2	119.3 (3)	C24—C23—H23A	119.9
C12—C11—C10	121.4 (3)	C22—C23—H23A	119.9
F2—C11—C10	119.3 (3)	C23—C24—C25	119.8 (4)
C11—C12—C13	119.0 (3)	C23—C24—H24A	120.1
C11—C12—H12A	120.5	C25—C24—H24A	120.1
C13—C12—H12A	120.5	C20—C25—C24	120.0 (3)
C12—C13—C8	121.9 (3)	C20—C25—H25A	120.0
C12—C13—H13A	119.1	C24—C25—H25A	120.0
C8—C13—H13A	119.1	C21—C26—H26A	109.5
N1—C14—C15	110.5 (2)	C21—C26—H26B	109.5
N1—C14—H14A	109.5	H26A—C26—H26B	109.5
C15—C14—H14A	109.5	C21—C26—H26C	109.5
N1—C14—H14B	109.5	H26A—C26—H26C	109.5
C15—C14—H14B	109.5	H26B—C26—H26C	109.5
H14A—C14—H14B	108.1		
			
C6—C1—C2—C3	−0.8 (4)	C9—C8—C13—C12	1.3 (4)
C1—C2—C3—C4	−1.5 (5)	C7—C8—C13—C12	179.9 (2)
C1—C2—C3—F1	178.9 (2)	C17—N1—C14—C15	−59.2 (3)
C2—C3—C4—C5	2.2 (5)	C7—N1—C14—C15	178.8 (2)
F1—C3—C4—C5	−178.2 (3)	C18—N2—C15—C14	179.8 (3)
C3—C4—C5—C6	−0.5 (5)	C16—N2—C15—C14	−56.4 (3)
C4—C5—C6—C1	−1.6 (4)	N1—C14—C15—N2	58.9 (3)
C4—C5—C6—C7	−179.6 (3)	C15—N2—C16—C17	57.3 (3)
C2—C1—C6—C5	2.3 (4)	C18—N2—C16—C17	−179.3 (3)
C2—C1—C6—C7	−179.8 (2)	C14—N1—C17—C16	60.6 (3)
C17—N1—C7—C8	−178.1 (2)	C7—N1—C17—C16	−177.5 (2)
C14—N1—C7—C8	−58.6 (3)	N2—C16—C17—N1	−61.3 (3)
C17—N1—C7—C6	58.5 (3)	C15—N2—C18—C19	−99.0 (3)
C14—N1—C7—C6	178.0 (2)	C16—N2—C18—C19	139.1 (3)
C5—C6—C7—N1	−139.5 (2)	C20—O—C19—C18	173.6 (3)
C1—C6—C7—N1	42.6 (3)	N2—C18—C19—O	66.0 (4)
C5—C6—C7—C8	96.5 (3)	C19—O—C20—C25	1.7 (4)
C1—C6—C7—C8	−81.3 (3)	C19—O—C20—C21	−178.2 (3)
N1—C7—C8—C9	−45.1 (4)	O—C20—C21—C22	178.3 (3)
C6—C7—C8—C9	78.7 (3)	C25—C20—C21—C22	−1.6 (4)
N1—C7—C8—C13	136.4 (2)	O—C20—C21—C26	−1.9 (4)
C6—C7—C8—C13	−99.7 (3)	C25—C20—C21—C26	178.2 (3)
C13—C8—C9—C10	−1.5 (5)	C20—C21—C22—C23	0.4 (5)
C7—C8—C9—C10	179.9 (3)	C26—C21—C22—C23	−179.4 (3)
C8—C9—C10—C11	0.9 (6)	C21—C22—C23—C24	0.4 (5)
C9—C10—C11—C12	0.1 (6)	C22—C23—C24—C25	0.0 (5)
C9—C10—C11—F2	−179.5 (3)	O—C20—C25—C24	−177.8 (3)
F2—C11—C12—C13	179.3 (3)	C21—C20—C25—C24	2.1 (5)
C10—C11—C12—C13	−0.3 (5)	C23—C24—C25—C20	−1.2 (5)
C11—C12—C13—C8	−0.4 (4)		
